# Lower p66Shc promoter methylation in subjects with chronic renal failure

**DOI:** 10.1371/journal.pone.0257176

**Published:** 2021-09-16

**Authors:** Radhia Hamdi, Amana Saadallah-Kallel, Slima Ferchichi-Trimeche, Raja Mokdad-Gargouri, Abdelhedi Miled, Bachir Benarba

**Affiliations:** 1 Faculty of Pharmacy, Research unit of clinical and molecular biology (UR17ES29), Department of biochemistry, University of Monastir, Monastir, Tunisia; 2 Laboratory of Biochemistry, Sidi Bouzid Regional Hospital, Sidi Bouzid, Tunisia; 3 Laboratory of Molecular Biotechnology of Eukaryotes, Biotechnology Center, University of Sfax, Sfax, Tunisia; 4 CHU Research Unit, Farhat Hached, Sousse, Tunisia; 5 Faculty of Nature and Life, Laboratory Research on Biological Systems and Geomatics, University of Mascara, Mascara, Algeria; University of Messina, ITALY

## Abstract

**Objective:**

To determine the correlation between DNA methylation of p66Shc promoter and some markers of inflammatory and oxidative stress in chronic renal failure (CRF) patients compared with healthy subjects.

**Methods:**

An observational cross-sectional study was conducted in the nephrology department at Sidi Bouzid Regional Hospital (Tunisia). In total, 39 patients with CRF and 37 healthy subjects were included. Several biochemical parameters were measured. Furthermore, markers of the oxidative and inflammatory status (MDA, TAS, SOD, and CRP) were evaluated. The p66Shc methylation status was determined using the methylation-specific PCR.

**Results:**

Our results showed that levels of blood glucose, urea, creatinine, uric acid, ChT, TG, albuminuria, CRP and MDA were significantly elevated in CRF patients compared to controls. Furthermore, p66Shc promoter region was highly demethylated in CRF patients compared to healthy controls (84% vs 4%). Our data showed a positive correlation between p66Shc hypomethylation and levels of MDA (r = 0.93; p<0, 05) and CRP (r = 0.89; P <0, 05), as well as a significant negative correlation between p66Shc hypomethylation, TAS (r = -0.76; P <0, 05) and SOD (r = -0.77; p<0, 05) levels. Similarly, there was a positive correlation between p66Shc hypomethylation and the disease stages. Importantly, multiple regression analysis showed that p66shc DNA hypomethylation remains strongly correlated with MDA, CRP and stages of CRF.

**Conclusion:**

This study indicates that the DNA hypomethylation of p66shc promoter was correlated with oxidative and inflammatory stress and the disease stages in CRF patients.

## 1. Introduction

Chronic renal failure (CRF) represents a public health issue requiring cumbersome management due to its increasing incidence, its prevalence, its chronic nature and the economic cost of its management [1]. Chronic renal failure is a complex, multifactorial and non-trans-generational pathology [2] resulting in renal structural changes, parenchymal damage and altered glomerular filtration rate [3,4]. Nowadays, the biological and clinical exploratory methods including factors risk, enzymatic assays, and genetic factors remain rather superficial and summary to explain the different pathways involved in the initiation of the renal pathology [5–7]. Recently, a large number of studies have demonstrated the association between chronic renal failure and oxidative stress. Production of ROS mainly by the mitochondrial respiratory chain, NADPH oxidase system, oxidases and uncoupling of nitric oxide synthase (NOS) is considered the main pathogenic agents of the disease [8–10]. In fact, it has been reported that the oxidative stress alters the renal functions in all nephron segments, and contributes to the initiation and promotion of renal injury in patients [11–13]. Oxidative stress has been shown to activate several cell signaling pathways that have deleterious effects on the cell growth by inducing cell death via necrosis or apoptosis [14–16]. Among these signaling pathways, that of Shc adaptor proteins family regulates different cellular functions [16]. Three Shc iso-forms are coded by the ShcA locus and are named according to their molecular weights, the protein Shc46 (p46 Shc), the protein Shc 52 (p52Shc) and the protein Shc66 (p66Shc). These proteins are involved in proliferation and apoptosis regulation [17]. Besides targeting Ras proteins and MAPK pathways, p66Shc has been shown to play an important role in ROS production owing to its additional CH2 domain containing a phosphorylated serine residue at position 36 in response to various environmental stimuli such as UV rays, toxic agents and diet [14–16]. Indeed, treatment of 293A human embryonic cells with high doses of glucose-induced over-expression of p66Shc, and did not affect the p46Shc and p52Shc expression [18]. Several studies have demonstrated that p66Shc possesses a redox activity and is involved in enhanced ROS production in response to different stimuli [19,20]. Over-expression of p66Shc causes excessive production of ROS, which in turn results in oxidative damage at the molecular, cellular and tissue levels [14,15]. In addition, inhibition of p66Shc in p66Shc (-/-) double- mutant mice resulted in improved resistance to oxidative stress, longer life, and decreased ROS-induced apoptosis [16]. These literature reports certainly establish the role of p66shc in the genesis of oxidant stress. However, the role and the details of epigenetic mechanisms involved in its regulation remain very limited. Epigenetics is a biological process that linking genetic and the environment, and its dysregulation is involved in the pathophysiology of many complex diseases including, chronic renal failure [2,5].DNA methylation is an extensively explored epigenetic phenomenon, that is often associated with histone modifications, chromatin conformation and RNA interference, all of which are processes that regulate gene transcription during embryonic and subsequent development [21]. Since the value of DNA methylation in the clinic is largely unknown and therefore studying DNA methylation is novel and could be of interest for patients with CRF. The main objective of the present study was to investigate the correlation between the DNA methylation level of p66Shc promoter and some markers of inflammatory and oxidative stress in CRF patients compared with healthy control subjects.

## 2. Subjects and methods

### 2.1. Patient selection

An observational cross-sectional study was conducted in the nephrology department at Sidi Bouzid Regional Hospital (Tunisia). A total of 76 subjects were recruited into the present study including 39 patients with chronic renal failure (cases) and 37 healthy subjects (control). The subjects included in the study (cases and controls), lived in the same region and did not report any supplementation with antioxidants. Subjects with recent viral or bacterial infection, HIV serotype, hepatitis B and hepatitis C were excluded from the study.

The controls (n = 37 including 19 men and 18 women) were free from all renal pathologies. The age of the controls was 59 ± 9 years (Min = 30, Max = 66 years).

Cases with chronic renal failure (n = 39 of which 20 men and 19 women) were aged 63 ± 11 years (Min = 23, Max = 78 years). This group was subdivided into three subgroups according to the stage of renal failure defined by the modification of diet in renal diseases (MDRD) formula [6]: GFR = 186 × (Crea× 0.0113)^-1,154^ × age^-0,203^× K.

Where

**K**: Correction factor = 0.742 for women and 1 for men
**Crea**: Plasma creatinine
  - Stage II: 13 patients (7 men and 6 women) representing 33% of cases;
  - Stage III: 11 patients (6 men and 5 women) constituting 28% of cases;
  - Stage IV: 15 patients (8 men and 7 women), ie 38% of cases

We excluded stage I patients since (being a beginner stage) they show only a few relatively mild and nonspecific changes. Likewise, patients in stage V were excluded from the study because, being in the terminal stage, they are treated with hemodialysis, which is often associated with various complications in addition to chronic renal failure.

The cases included in the study had chronic renal failure with different etiologies. 31% of patients had diabetic nephropathy, 14% had a polycystic disease, 37% suffered from glomerulosclerosis, 3% had family factors, and 15% had unknown etiology.

### 2.2 Samples collection

Blood samples were collected by venepuncture from subjects with a minimum of 12 hrs of fasting. After centrifugation, plasma samples were stored at -80° C until analyses.

#### i. Biochemical measurements

Plasma glucose levels, Urea, creatinine, uric acid, total cholesterol (TC), high-density lipoprotein cholesterol (C_HDL), and triglycerides (TG) were measured by standardized enzymatic colorimetric method using an automated system (Beckman coulter), following the instructions of the manufacturer (Randox-Antrim, UK).

C-reactive protein (CRP), a marker of inflammatory status, was measured using a latex-enhanced turbidimetric immune assay following instructions of the manufacturer (Randox-Antrim, UK). Human CRP binds to latex particles coated with monoclonal anti-CRP antibodies. The haze is measured by turbidimetry at 340 nm.

The level of albuminuria was determined in fresh urine using the immunoturbidimetric method (Randox-Antrim, UK).

Malondialdehyde (MDA) was determined using the thiobarbituric acid method [22,23]. In brief, 150 μl of plasma and 50 μl of bidistilled water were incubated at 60°C for 30 minutes. 500 μl of 20% trichloroacetic acid were then added and the mixture was centrifuged at 3000 g for 5 minutes. 500 μl of the supernatant were mixed with 250 μl of 0.8% thiobarbituric acid for 30 minutes in a boiling water bath (100°c). The absorbance of the pink supernatant was measured at 532 nm.

The antioxidant enzyme Mn superoxide dismutase (SODMn) activity was determined by the method described by Fridovich [24] following the manufacturer instructions (Antrim, Uk). In this method xanthine and xanthine oxidase are employed to generate uric acid and superoxide anion. The latter reacts with 2- (4-iodopheny1) -3- (4-nitropheno1) -5-phenyltetrazolium chloride (INT), and the resulting red formazan dye was measured at 505 nm.

The total antioxidant status (TAS) was determined by an enzymatic method using the Randox Laboratories kit (Antrim, Uk). In this assay, 2,2’-Azino-di- [3-ethylbenzthiazoline sulphonate] incubated in the presence of H_2_O_2_ and peroxidase, gives rise to a cationic radical ABTS produce cationic radical ABTS^+^, a relatively stable blue-green compound absorbing at 600nm. The antioxidants present in the sample added induce a reduction in the production of this compound in proportion to their concentration.

#### ii. DNA extraction

The salting out method was used to extract the genomic DNA from the whole blood. Extracted DNA was quantified by measuring the absorbance at 260 nm using a spectrophotometer (NanoDrop-1000). The purity of the extracted DNA was assessed through the ratio of UV-absorbance at 260 nm and the absorbance at 280 nm (A260/A280). A ratio of 1.7–2 indicates that DNA preparation is pure and not contaminated.

#### iii. P66Shc methylation status

The methylation status of p66Shc was determined using the methylation-specific PCR (MSP). The latter requires modification of the genomic DNA using the sodium bisulfite modification technique, according to the "ZDNA Methylation ™" kit protocol (ZYMO Research). This technique known as "the gold standard" is widely used [25].

The expression of p66Shc was determined using:

- One pair of methylated "M" primer: one for sense (MS): (5’-AGT TTT ATT TT TGT TT AGT TCG A-3 ’) and the other antisense (MR): (5’- CTA ACTCCT CCC CAA AAA CG -3 ’). It was designed by the Met primer software, with a temperature Tm of 55.4°C chosen with optimase protocol writer to amplify a specific region of 171 pair of base (pb) of the p66Shc promoter region.
- One pair of "U" primers: one for sense (US): (5’-TGG ATA GTT TTA TT TT GTT TTA GTT TG-3 ’) and the other antisense (UR): (5’-TTC TAA CTC CTC CCC AAA AAC A -3 ’), with a temperature Tm of 53°C, determined by the optimase protocol writer and designed by the Met primer software to amplify a 170 bp specific sequence to the p66Shc promoter region.

After optimization, the PSM mix is constituted of a final volume of 25 μl including18.8 μl of bi-distilled water, 2 μl of dream buffer, 2.5μl of MgSO_4_, 0.5μl of DNTP, 0.5μl of sense and antisense primers, and 0.2μl of Taq—DNA polymerase (Dream Taq) Fermentas.

Amplification was carried out as follows: after initial denaturation (94°C/5min), 35 amplification cycles were carried out. Each cycle consisted of a denaturation (94°C/30 Sec) followed by hybridization of the primers on DNA (53°C/30 sec for primer U and 55.4°C/30 sec for primer M), an extension at 72°C for 30 seconds and a final extension for 5 min. Sterile bidistilled water was used as a control.

The methylation-specific PCR products were identified by ethidium bromide staining after 2.5% agarose gel electrophoresis (10X TBE buffer; 100 Volt). Visualization was carried out using a Kodak 1D image analysis system (GelDoc).

The p66Shc methylation status was evaluated by the score S, which is dependent on the intensity of the fluorescence and can, therefore, be determined as a percentage of methylation for each patient. S = 0 corresponds to hypermethylation, S = 0.5 corresponds to a hemimethylated state and S = 1 corresponds to hypomethylation.

To evaluate the persistent relation between oxidative and inflammatory stress and p66shc DNA hypomethylation, consecutive weekly measurements of MDA, CRP and methylation-specific PCR were performed during 2 months in CRF patients.

### 2.3. Statistical analysis

Statistical analyses were performed with SPSS version 22.0. Normality distribution of the data was examined by the Shapiro-Wilk test.

The statistical comparisons of independent samples were made by one-way ANOVA parametric test followed by non-parametric Mann-Whitney and Kruskal Wallis Test U tests.

The proportions between independent samples were compared by the Chi-square test.

Correlation analyses were evaluated by the linear relationship and the Spearman correlation.

Regression and multiple regression analyses were carried out.

Statistical significance was defined as p< 0.05.

### 2.4. Ethics approval

The study was performed based on standard principles of ethical and professional conduct and was approved by the Ethics Committees of University Hospital Farhat Hached Sousse (Note: N°1/2019). All participants were informed that their data and their blood samples will be used for the research and all have signed an informed consent.

## 3. Results

**Fig 1:**
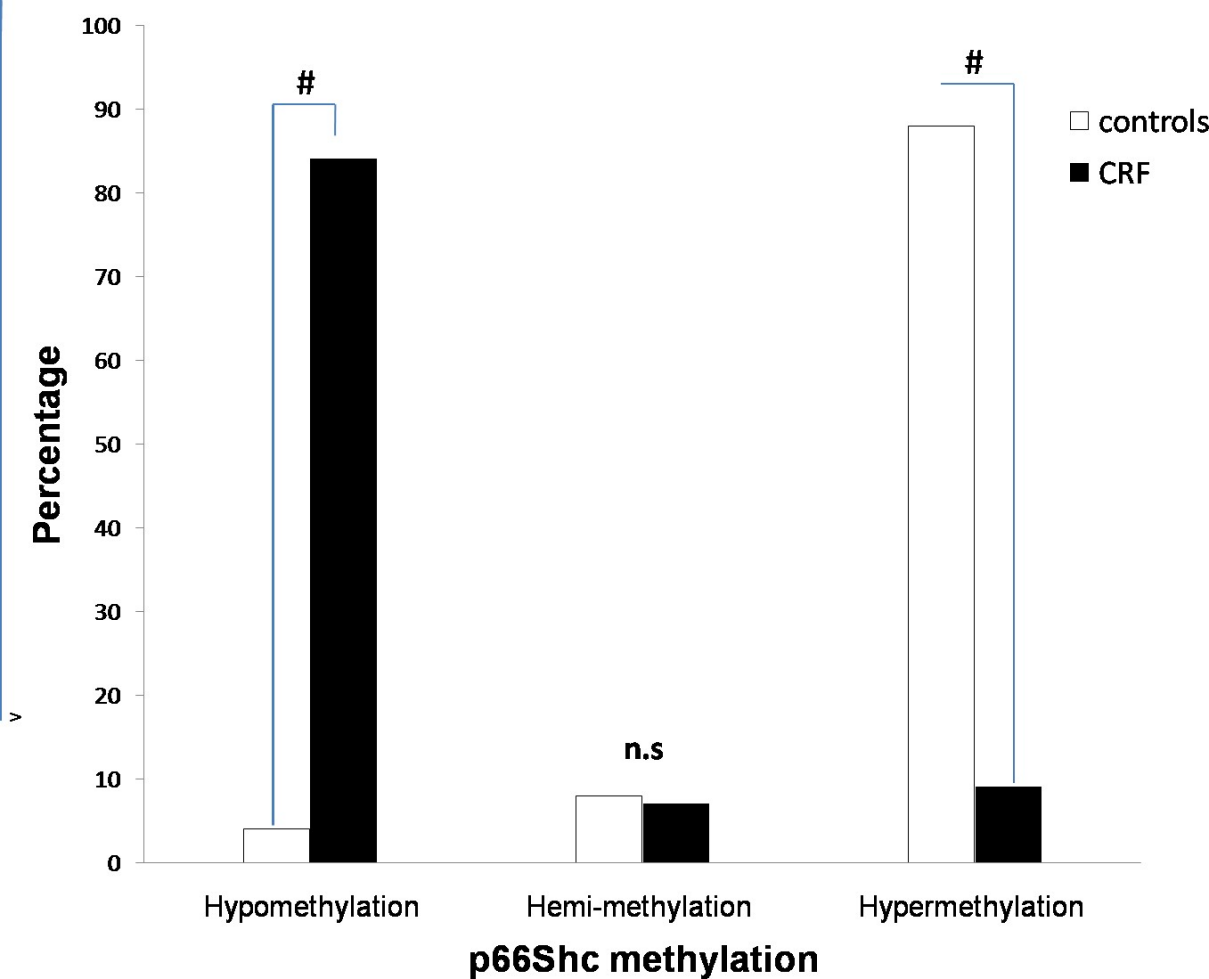
DNA methylation state of p66Shc promoter in CRF patients and controls.

Regarding methylation of p66Shc promoter, our data (Fig 1) showed that CRF patients were characterized by an increased p66Shc hypomethylation (84%) as compared to controls (4%). In contrast, a significant increased p66Shc hypermethylation was detected in controls patients (88% *vs* 9% in CRF). However, no significant difference in p66Shc hemi-methylation was found for the two groups (8%in controls vs 7% in CRF).

As shown in Table 1, when CRF patients and controls were compared according to the sex, age, smoking, and arterial hypertension, no significant differences were found. However, we found a significant increase in the prevalence of diabetes (31%) in patients with chronic renal failure compared with controls. Furthermore, levels of blood glucose, urea, creatinine, uric acid, ChT, TG, albuminuria, CRP and MDA were significantly elevated in CRF patients when compared with controls. In addition, the levels of blood markers (urea, creatinine, uric, CRP and MDA) increased considerably with advancing stages of chronic renal failure. On the other hand, we observed a significant decrease in Hb, HDL-C, TAS and SOD levels in the cases compared to controls. Markedly, the reduction of SOD and TAS levels was more significant with the disease stages (Table 2).

**Table 1:** Clinical characteristics, renal function, inflammatory and oxidative stress biomarkers and DNA p66shc methylation markers in CRF and controls patients.

Variable	controls (n = 37)	CRF (n = 39)	P
Age(y)	59.00±9.00	63.00±11.00	0,063
Sex (m/f)	19/18	20/19	0,073
Smoking (%)	29%	33%	0,067
Diabetes (%)	11%	31%	0.0001
HT (%)	23%	27%	0,061
Alb (g/l)	0,13	1.35±1.91	0.0001
Hb (g/l)	13.83±0.98	11.80±1.00	0.0001
Glucose(mmol/l)	4.60±0.60	7.72±3.55	0.0001
BUN (mmol/l)	5.00±1.34	112.75±1.70	0.0001
Creatinine(μmmol)	73.72±10.89	198.94±13.95	0.0001
Uric acid (μmol/l)	276.93±37.19	387.50±86.75	0.0001
Total cholesterol (mmol/l)	4.73±0.98	7.45±1.16	0.0001
Triglycerides (mmol/l)	1.74±0.34	5.25±1.52	0.0001
HDL-cholesterol (mmol/l)	1.36±0.25	0.87±0.13	0.0001
MDA (μmol/l)	1.05±0.28^a^	3.39±1.18^b^	0.0001
CRP(mg/l)	2.65±1.41^a^	10.07±2.73^b^	0.0001
TAS (mmol/l)	1.52±0.12	1.07±0.17	0.0001
SODMn (U/gHb)	1195.86±339.11	905.72±153.47	0.0001
Hypomethylation (%)	4^a^	84^a^	0.0001

^a^Based on one methylation-specific PCR,MDA and CRP measurement.

^b^Based on the mean of weekly MDA and CRP measurements during two months.

**Abbreviations**: y: Years; m: Male; f: Female; N.S: Non-significant result. Alb: Albuminuria, HDL: High-density lipoprotein; BUN: Blood urea nitrogen; HB: Hemoglobin; HT: Hypertension, MDA: Malondialdehyde; CRP: Protein C Reactive; TAS: Total antioxidant status, SOD: Superoxide Dismutase, Mn: Magnesium.

**Table 2:** Clinical characteristics, renal function, inflammatory and oxidative stress, and DNA methylation markers levels according to CRF stages.

Variable	Stage II (13)	Stage III (11)	Stage IV (15)	Signification (P)
Age(y)	59.10±5.03	63.25±7.15	64.47±6.81	0,063
Sex(m/f)	07/6	6/5	8/7	0,066
Smoking (%)	35	33	32	0,070
Diabetes (%)	36	31	33	0,069
HT (SBP/DBP) (mmHg)	143.00±3.50/75.00±2.30	144.00±4.80/74±3.40	142±5.2/81±3.6	0,077
Alb (g/l)	1.85±1.91	1.93±1.22	2.04±1.78	0,065
Glucose(mmol/l)	6.61±0.61	7,79±0.38	8,531±0.54	0,072
BUN(mmol/l)	9.37±1.34	10.80±1.70	11.90±2.90	P^1,2,3^
Creatinine(μmmol/l)	178.07±56.22	198.94±64.76	211.54±78.23	P^1,2,3^
Hb (g/l)	11.58±1.25	11.56±1.12	10.87±1.78	0,065
Uric acid (umol/l)	361.76±75.20	389.75±86.57	408.23±87.03	P^1,2,3^
Total cholesterol (mmol/l)	6.88±1.36	6.51±1.53	6.65±1.79	0,066
Triglycerides (mmol/l)	3.88±1.12	5.88±1.65	6.01±1.65	0,063
HDL-cholesterol (mmol/l)	0. 86±0.25	0.83±0.89	0.89±0.13	0,067
MDA (μmol/l)	2.90±1.18^b^	3.49±1.46^b^	3.86±1.28^b^	P^1,2,3^
CRP (mg/l)	8.81±2.73^b^	9.92±1.14^b^	11.48±2.63^b^	P^1,2,3^
TAS (mmol/l)	1.12±0.12	1.08±0.13	0.91±0.27	P^1,2,3^
SOD (U/gHb)	1001.23±23.80	915.30±30.60	803.22±228.00	P^1,2,3^
Hypomethylation p66shc (%)	19,24^b^	33,48^b^	47,29^b^	P^1,2,3^

P^1^ < 0.001: Stage II *vs* Stage III.

P^2^ < 0.001: Stage II *vs* Stage IV.

P^3^ < 0.001: Stage III *vs* Stage IV.

Abbreviations are as shown in Table 1.

SBP: Systolic blood pressure.

DBP: Diastolic blood pressure.

^b^Based on the mean of weekly methylation-specific PCR,MDA and CRP measurements during two months.

As shown in (Table 2), MDA concentrations increased significantly with advancing stages of chronic renal failure, from 2.9±1.1 (stage II) to 3.86±1.2 μmol/l (stage IV).

Regarding the variation of the inflammatory and the antioxidant status with the disease stages, our results showed a significant increase of CRP levels from 8.81±2.73 in stage II to 9.92±1.1 and 11.48±2.63 mg/ml in stages III and IV, respectively. On the other hand, SOD and TAS levels decreased significantly with advancing of the disease stages. Indeed, TAS decreased from 1.12±0.12 in stage II to 0.91±0.27 mmol/l in stage IV, whereas SOD levels were reduced from 1001.23±238 in stage II to 915.30±306 and 803.228±228 U/gHb in stages III and IV, respectively.

Markedly, a strong positive correlation between MDA levels and diabetes in CRF patients was observed (P = 0, 0001, r = 0, 84), whereas no significant correlations were found between MDA levels and, age, sex, smoking and HT in CRF patients. Furthermore, our data demonstrated a positive correlation between MDA, total cholesterol (r = 0.69), blood glucose (0.81), and CRP (r = 0.87), and on the other hand, a strong significant negative correlation with TAS (r = -0.86). A moderate significant negative correlation (r = -0.56) with SOD and MDA was found (Table 3).

**Table 3:** Correlation between MDA levels and some parameters in CRF patients by univariate analysis.

MDA (μmol/l)^b^		
	P	r
Sex(m/f)	0.43	0.27
Smoking (%)	0.47	0.39
Age(y)	0.31	0.29
HT (%)	0.3 3	0.23
Diabetes (%)	0,0001	0,84
Glucose(mmol/l)	0.0001	0.81
Total cholesterol (mmol/l)	0.0001	0.69
CRP(mg/l)^b^	0.0001	0.87
TAS (mmol/l)	0.0001	-0.86
SODMn (U/gHb)	0.02	-0.56

Abbreviations are as shown in Table 1.

^b^Based on the mean of weekly methylation-specific PCR,MDA and CRP measurements during two months.

As highlighted in Table 4, no significant correlation between p66Shc DNA hypomethylation and, age, sex, smoking, hypertension, albuminuria, Hb and HDL-Cholesterol has been found. However, our results showed a positive correlation between p66ShcDNA hypomethylation and diabetes (r = 0.79; *P* = 0.0001). Similarly, the p66Shc hypomethylation state was positively correlated with the elevated levels of blood glucose (r = 0.88; *P* = 0.0001), total cholesterol (r = 0.71; *P* = 0.003) and TG (r = 0.70; *P* = 0.001). Besides, a positive correlation between p66Shc hypomethylation and, urea (r = 0, 69, p = 0,001), creatinine (r = 0, 67, p = 0,001), and uric acid (r = 0, 69, p = 0,002) was demonstrated (Table 4).

**Table 4:** Correlation between p66Shc DNA hypomethylation and clinical characteristics, inflammation and oxidative stress biomarkers in CRF subjects by univariate analysis.

	p66Shc hypomethylation^b^	
	P	r
Sex(m/f)	0,081	0.25
Smoking (%)	0,079	0.31
Age(y)	0,080	0.26
Diabetes (%)	0.0001	0,79
HTA (%)	0,069	0.39
Stages of CRF	0,0001	0,87
Alb (g/l)	0,076	0.32
Glucose(mmol/l)	0.0001	0.88
Hb(g/l)	0,078	0,29
Total cholesterol (mmol/l)	0.003	0.71
BUN(mmol/l)	0.001	0.69
Creatinine(μmmol)	0.001	0.67
Uric acid (umol/l)	0.002	0.69
HDL-cholesterol (mmol/l)	0,067	0,36
Triglycerides (mmol/l)	0,001	0,70
MDA(μmol/l)^b^	0,0001	0,93
TAS(mmol/l)	0,0001	-0,76
SOD(U/gHb)	0,0001	-0,77
CRP(mg/l)^b^	0,0001	0,89

Abbreviations are as shown in Table 1.

^b^Based on the mean of weekly methylation-specific PCR,MDA and CRP measurements during two months.

As shown in Table 4, the p66Shc hypomethylation increased significantly with MDA and CRP levels in subjects with chronic renal failure. Moreover, there was a significant decrease in SOD, and TAS levels with p66Shc hypomethylation. Importantly, the correlation analysis showed a positive correlation between p66Shc hypomethylation and levels of MDA (r = 0.93; *P* = 0.0001) and CRP (r = 0.89; *P* = 0.0001), as well as a significant negative correlation between p66Shc hypomethylation, TAS (r = -0.76; *P* = 0.0001) and SODMn (r = -0.77; *P* = 0.0001) levels. On the other hand, there was a positive correlation between p66Shc hypomethylation and the disease stages (r = 0.87; *P* = 0.0001) was observed (Table 4).

When we performed a multivariate regression analysis (Table 5), including only the variables that were significant in the univariate analysis, p66Shc DNA hypomethylation remained strongly correlated with both MDA (r = 0,91,*P* = 0.0001), CRP (r = 0,71,*P* = 0.0001) and stages of CRF(r = 0,79, *P* = 0.0001), whereas all other variables lost their association with outcome.

**Table 5:** Correlation between p66Shc DNA hypomethylation with clinical characteristics, inflammation and oxidative stress biomarkers in CRF patients by multiple analyses.

	p66Shc hypomethylation^b^	
	P	r
Diabetes (%)	0,060	0,49
Stages of CRF	0,0001	0.79
Glucose(mmol/l)	0,063	0.48
Total cholesterol (mmol/l)	0,072	0.33
BUN(mmol/l)	0,083	0.31
Creatinine(μmmol)	0,091	0.27
Uric acid (umol/l)	0,069	0.39
Triglycerides (mmol/l)	0,087	0,35
MDA(μmol/l)^b^	0,0001	0,91
TAS (mmol/l)	0,061	0,46
SOD(U/gHb)	0,089	0,29
CRP(mg/l)^b^	0,0001	0,71

Abbreviations are as shown in Table 1.

^b^Based on the mean of weekly methylation-specific PCR,MDA and CRP measurements during two months.

## 4. Discussion

Chronic renal failure considered a public multifactorial issue, is regulated by epigenetic modifications. The disease is characterized by stimulation of certain signaling pathways and transcription factors of pro-oxidative and pro-inflammatory genes following exposure to certain environmental factors. Lifestyle and exposure to environmental risk factors could negatively affect the regulation of the cellular epigenome [26]. DNA methylation is an epigenetic phenomenon, varies in response to the change in environment cellular. Notably, methylation at promoter region could influence the gene expression at the transcription level, in which highly-methylated promoter region could suppress gene expression and vice versa [27].

In the present study, we found a significant positive correlation between DNA hypomethylation level of p66Shc promoter and inflammatory and oxidative stress as well as some clinical and biological characteristics in patients with chronic renal failure compared to controls.

Our results showed a significant positive correlation between p66Shc DNA hypomethylation and high glucose level in subjects with chronic renal failure compared to controls. These findings are consistent in partly with those previously reported. It has been demonstrated that hyperglycemia induced over-expression of p66Shc through hypomethylation and histone3 acetylation (H3 ac), mainly as a result of methyltransferase DNMT3b and histone deacetylase SIRT1 down regulation [28]. Indeed, Paneni et al [29] reported similar findings in endothelial cells (Ecs) exposed to high glucose and in aortas of diabetic mice, in which overexpression of p66Shc is epigenetically regulated by its promoter CpG hypomethylation and by GCN 5-induced histone 3 acetylation. Moreover, Wang et al [30] found that increased glucose concentrations enhanced p66Shc expression. Consequently, p66shc has been suggested to be used as a marker of diabetic nephropathy [31]. Our results showed a significant positive correlation between DNA hypomethylation of p66Shc promoter in diabetic’s subjects with chronic renal failure compared to control. Further studies are needed to elucidate the mechanism through which diabetes may mediate the DNA hypomethylation of p66Shc promoter related with CRF.

The present study revealed a positive correlation between DNA hypomethylation of p66Shc promoter and high levels of MDA, as well as a significant negative correlation between p66Shc hypomethylation, TAS and SODMn levels in CRF patients. Since MDA is a surrogate marker of oxidative stress and both SOD and TAS are parameters reflecting antioxidant system [32], here we show that p66Shc hypomethylation is positively related with oxidative stress in CRF patients. Renal failure is closely related to oxidative stress, and due to the ability of p66Shc to reflect its sensitivity [33], a better understanding of the pathways regulating its expression could be useful in clarifying the pathophysiology of chronic renal failure. Although the p66shc promoter is not strongly methylated in human umbilical vein endothelial cells (HUVEC), demethylation of only a few CpGs [5,6] could affect gene expression [34]. It has been demonstrated that p66shc hypomethylation may exert an effect on its gene expression [28–30]. A positive correlation between MDA and p66Shc expression has been previously demonstrated [15]. Furthermore, several studies have demonstrated that p66Shc expression is related to mitochondrial dysfunction, enhanced ROS generation and oxidative damage [35–37]. Using multivariate analysis, our data showed that DNA hypomethylation of p66Shc promoter in PBL remained strongly correlated with the high levels of MDA and the stages of disease in CRF. It has been shown that p66Shc gene was involved in oxidative stress pathways [15,16]. Since MDA is the end product of lipid peroxidation used as a surrogate marker of oxidative stress [32], the potential correlation between high MDA levels and DNA hypomethylation of p66Shc promoter could negatively affect the p66Shc promoter methylation process. Further in -depth studies are needed to understand the mechanism related to the decreased DNA methylation of p66Shc promoter associated with CRF. It has been suggested, that p66Shc possesses a redox activity, both through mitochondrial ROS over-production, and decreased expression of antioxidant enzymes such as SOD [17,38,39]. Our results showed a significant decrease in SODMn levels in CRF patients compared with controls. Moreover, SODMn levels decreased significantly with advancing of the disease stages. It is important to note that SODMn levels were negatively correlated with DNA hypomethylation of p66Shc promoter. It has been reported that p66shc can increase mitochondrial H_2_O_2_ production and inhibit FoxO_3_a activity [40]. Actually, p66Shc by acting as a redox enzyme in the mitochondrion inhibits FOXO3a, a transcription factor that activates the synthesis of antioxidant enzymes such as SOD [40]. In addition, it was found that the ROS overproduction (mediated by p66Shc following stimulation by hyperglycemia) activated AKT1/PKb kinases, resulting in phosphorylation and inactivation of the FOXO3a protein [18,38], and also inhibition of SOD expression by increased recruitment of repressive epigenetic markers (H4K20me (3)/SUV420H2) in its promoter [38]. On the other hand, it is thought that the high level of p66Shc reduces the levels of antioxidant enzymes (Glutathione peroxidase-1, SOD) and transcription factors (FOXO3a/RF-1) responsible for their expression [17,18,38]. According to our results, a negative correlation between SOD levels, DNA hypomethylation of p66Shc and high levels of MDA was found in CRF patients. Besides, our data showed that the TAS levels were significantly lowered in subjects with chronic renal failure. A negative correlation was found between TAS and MDA, and between TAS and DNA hypomethylation of p66Shc promoter in these subjects according to stages diseases. Thus, persistent oxidative stress demonstrated in CRF patients may cause aberrant DNA methylation of p66shc promoter, via a number of possible mechanisms. Clearly, further studies are needed to resolve the complex interactions between oxidative stress and p66shc DNA hypomethylation in CRF patients.

It has been suggested that CRF is closely related to oxidative and inflammatory stress [25,41,42]. We therefore evaluated the association between inflammatory and oxidative status. Our results showed a positive correlation between CRP and MDA, and between CRP and DNA hypometylation of p66Shc promoter. Importantly, the CRP levels increased significantly with stages of the disease, which could be attributed to p66shc-induced ROS overproduction leading to activation of pro-inflammatory transcription factors by epigenetic modifications [43]. Indeed, it has been found that ROS can induce pro-inflammatory-related molecular and genes expression processes through activation of the redox-sensitive transcription factor, NFκB [34,38,43,44]. Moreover, it has been reported that oxidative stress can also activate inflammatory cytokines, especially in CRF patients with diabetes and hypertension [26,41]. IL-6 is the primary trigger for liver cells to synthesize C-reactive protein (CRP), in which both IL-6 and CRP have been shown to be at higher levels in CRF patients [41]. Therefore, these inflammatory molecules may potentially contribute to alter the p66shc gene expression in monocytes, leading to a damaging vicious cycle in CRF patients [38,43]. The positive correlation CRP-MDA could suggest the presence of an association between oxidative status and inflammation in CRF patients. A similar positive correlation between CRP and MDA has been previously reported [45,46]. In fact, the oxidative and inflammatory situations are strongly associated with chronic renal failure and have a synergistic effect leading to permanent stress responsible for the initiation and progression of the disease [41,47,48]. The persistent strong correlation between CRP and, MDA, stages of CRF and DNA hypometylation of p66Shc promoter could provide a valuable insight in understanding the molecular basis of CRF and could be extended to further in-depth molecular analysis. CRF patients enrolled in our study also had associated imbalanced diet, dyslipidemia and pharmacological treatment. We cannot rule out potential interfering effects of these covariables as well as other very complex individual exposures on our results.

Overall, the present study give evidence that DNA hypomethylation of p66shc promoter is positively correlated with oxidative and inflammatory status, as well as CRF stages. Furthermore, DNA hypomethylation of p66shc promoter is positively correlated with some clinical and biological characteristics in CRF patients.

### Limitations and future direction

Despite the importance of this work, the findings are limited due to the cross-sectional nature of the study. Thus, future research should include the longitudinal observational study approach to investigate the correlation between DNA methylation level and gene expression level over- time in CRF patients. In this study, the DNA methylation of p66shc promoter was measured in the peripheral leucocytes by a non-invasive method, unlike to renal tissues.

Although there have been discrepancies in the use of peripheral tissue to reflect changes in target tissues, recent attempts have investigated the feasibility of using blood as a surrogate tissue for other inaccessible ones [49,50]. More in-depth studies are needed to further understand the use of methylation markers in the blood to reflect the corresponding profile in the target tissue. Knowing the correspondence between blood methylome and inaccessible tissues is vital for our future understanding.

## 5. Conclusions

The current study indicates that hypomethylation DNA of p66shc promoter was correlated with oxidative and inflammatory stress and CRF stages. Gene expression and longitudinal methylation studies are warranted to establish the methylation effect on p66shc- expression over time.

## Abbreviations

CRF: chronic renal failure
MDRD: modification of diet in renal diseases
NOS: nitric oxide
Ecs: Endothelial cells
HUVECs: Human umbilical vein endothelial cell
Ras: rat sarcoma viral oncogene
Shc: Src homology and collagen
MAPK: mitogen-activated protein kinases
ROS: reactive oxygen species
HIV: human immunodeficiency virus
UV: Ultra Violet rays
TC: total cholesterol
C_HDL: high-density lipoprotein cholesterol
TG: triglycerides
CRP: C-reactive protein
MDA: malonaldehyde
SODMn: manganse superoxide dismutase enzyme
TAS: total antioxidant status
PCR: polymerase chain reaction
SMP: methylation-specific PCR
Pb: pair of base
MS: methyleted "M" primer for sense
MR: methyleted "M" primer for antisense
US: Umethyleted "U" primer for sense
UR: Umethyleted "U" primer for antisense
TBE buffer: buffer Tris Borate EDTA
CH2: collagen homology domain
DNMT3b: DNA methyl transferase 3b
H3 ac: histone3 acetylation
SIRT1: histone deacetylase class III
H2O2: hydrogen peroxide
FoxO3a: Forkhead box O3 is a human protein
AKT/PKB: signaling pathway (protein kinase B/phosphatidyl linositol3-kinase)
H4K20me (3): tri-methylation lysine 20 histone 4
SUV420H2: methyl-transferase
RF-1: redox factor 1
HK-2 cells: renal proximal tubular cells
pkc-β: protein kinase Cβ
PBL: peripheral blood leucocytes
